# Correspondence

**Published:** 1874-08

**Authors:** A. G. Smythe

**Affiliations:** Baldwyn, Miss.


Baldwyn, Miss., July 13, 1874.

*Editors Southern Medical Record:*

In the March number of The Southern Medical Record, page 133, is an article by Dr. John H. Booth, on the *Average Weight of Children at Birth.*

From a long, and I might say, an extensive country practice and observation, I have come to the same conclusion, which he expresses in the first part of the first paragraph of the article: *i. e.*, That children born in the rural districts of America, are much heavier as a general thing than those born in European cities, or rather in the maternity hospitals of the crowded cities of Great Britain and the continent.

True it is, that in my practice, there has not been a regular record kept, nor have all the children born in my care been weighed; but a very large majority have been carefully weighed and noted by myself. All the very large, as well as the very small, children were carefully weighed, and the conclusion is, that one-third of the number weighed were above seven pounds, that one-fifth weighed eight pounds, and that one-seventh weighed ten pounds and upward. Only one, in two thousand and four hundred, weighed four pounds; all others were above five pounds; one wighed sixteen, one fifteen, two thirteen, and four weighed twelve and a half each; one-third of the number probably weighed less than seven pounds, say an average of six pounds. Upon summing up, I find that the foregoing estimation will make an average of seven pounds, two ounces, five drachms and a fraction, which has fallen below what I had generally supposed was the average weight of American children at birth.

Permit me to say on this point, that all the very large, as well as the very small, children were viable and did well. The small one is now nineteen years of age, a rather tall, light woman, but always well.

The cause of the difference of the weight of American rustic and European hospital children is twofold; first, the nature of the labor and exercise; and in the second place, the quality and quantity of the diet of American women; both causes giving a decided advantage in the development of American children.

A. G. SMYTHE, M.D.

